# The association between *PTPN22* C1858T gene polymorphism and type 1 diabetes mellitus: an Indonesian study

**DOI:** 10.1080/07853890.2023.2190162

**Published:** 2023-03-24

**Authors:** Nur Rochmah, Fatimah Arief, Muhammad Faizi, Sukmawati Basuki

**Affiliations:** a Department of Child Health, Faculty of Medicine, Dr. Soetomo General Hospital, Universitas Airlangga, Surabaya, Indonesia; b Institute of Tropical Disease, Universitas Airlangga, Surabaya, Indonesia; c Department of Medical Parasitology, Faculty of Medicine Universitas Airlangga, Dr. Soetomo General Academic Hospital, Surabaya, Indonesia

**Keywords:** Type 1 diabetes mellitus, *PTPN22*, children

## Abstract

**Background:**

Type 1 diabetes mellitus (T1DM) is disease caused by the destruction of β pancreatic cells. The activation of T-lymphocyte and proliferation inhibitor are induced by protein tyrosine phosphatase non-receptor type 22 (*PTPN22*). However, the link between *PTPN22* C1858T gene polymorphism and T1DM is still controversy. This study aimed to analyse the C1858T gene polymorphism in Indonesian children with T1DM.

**Materials and methods:**

This case-control study was conducted from March 2021 to May 2022 in the Endocrinology Outpatient Clinic at Dr. Soetomo Hospital and Tropical Disease Center Universitas Airlangga. Patients with controlled T1DM during the study period were included. The *PTPN22* analysis used polymerase chain reaction restriction fragment length polymorphism (PCR-RFLP) method.

**Results:**

Sixty-two children voluntarily participated in this study, and were equally divided into the T1DM and control groups. Most of the patients (94%, 58/62) are Javanese. This study revealed a more frequent CC genotype (9.4%) and allele-C (54.6%) polymorphism in the T1DM group, while more frequent CT genotype (100%) and allele-T (50%) polymorphism were in the control group. The C- and T-allele frequency was 54.6% and 45.4% in the T1DM group, respectively. The T1DM and control groups did not significantly differ (*p*= .2381).

**Conclusions:**

*PTPN22* homozygous genotype-CC and allele-C polymorphisms are more frequent in patients with T1DM. However, the *PTPN22* C1858T gene polymorphism did not significantly correlate to T1DM children in this study.

**Key Messages:**
1. The *PTPN22* C1858T gene polymorphism does not significantly affect the susceptibility of T1DM in Indonesian children.
2. *PTPN22* homozygous genotype-CC polymorphism was more observed in the T1DM group; thus, this genotype may play as a risk factor for T1DM children in the Indonesian population.

## Background

Diabetes mellitus (DM) is a metabolic disorder characterized by persistent hyperglycaemia. Two forms of DM frequently affect children; type 1 diabetes mellitus (T1DM) is commonly reported as an autoimmune illness due to β-pancreatic cell destruction, and type 2 DM (T2DM), also known as non-insulin dependent DM. Both types of diabetes are polygenic, which means they are linked to various genes [1].

The prevalence of T1DM was 10% from diabetic patient [2]. The prevalence of T1DM is <1% of the entire population, and its incidence rate rapidly increased globally, an estimated threefold increase in prevalence by 2040 [3]. T1DM incidence is very different in some places. Some studies have found that the prevalence of T1DM was lower among Asians than Caucasians [4–10]. China reported a T1DM annual incidence of 0.1/100,000, Japan with 1.4/100,000, and Finland with 43/100,000, while in Indonesia is approximately 0.3/100,000 [2].

There are various causes of T1DM, such as genetic risk factors including β-pancreatic cell destruction induced by T-cell [2,11,12]. In 2008, Ikegami et al. reported that Japanese and Korean populations had five new single-nucleotide polymorphisms (SNPs). The −1123G > C SNP was correlated with T1DM in both populations. The human leukocyte antigen (HLA), cytotoxic T lymphocyte antigen-4 (CTLA-4) and protein tyrosine phosphatase non-receptor type 22 (*PTPN22*) are the crucial genes associated with T1DM susceptibility [13]. In Indonesian study, the HLA-DQA1 and HLA-DQB1 subtypes mainly found in Indonesian children with T1DM are HLA-DQA1 0101/0102 and HLA-DQB1 0301 [14]. Furthermore, the CTLA-4 1822 C/T polymorphism might be a protective factor against T1DM [15].

The PTPN22 gene has a significant role in preventing T cell activation and proliferation. The mutation of this gene can induce and maintain the autoimmunity [16]. *PTPN22* polymorphism varies among races. Studies have investigated the relationship between T1DM and the *PTPN22* C1858T. There have been reports of the 1858T allele being linked to T1DM in several countries, including Italy, Germany, Spain, Ukraine and France [13]. This topic has been studied since 12 years ago [12,17,18]. However, *PTPN22* studies in Indonesia is limited. Hence, this study aims to evaluate the association between children with *PTPN22* C1858T gene polymorphism and T1DM.

## Materials and methods

Study was conducted in the Pediatric Endocrine Outpatient Clinic at Dr. Soetomo Hospital and Tropical Disease Center (TDC) Universitas Airlangga from March 2021 to May 2022. Children aged 4–18 years, who are willing to join in this study, were included in the T1DM group. Type 1 DM diagnosis was based on classic symptoms, elevated blood glucose level, low C-peptide and positive antibodies (GAD-65 and ZnT8) [19].

Meanwhile, children without T1DM, who visited the Pediatric Outpatient Clinic at Dr. Soetomo Hospital, in stable condition, and willing to join this study, belonged to the control group. Children due to severe illness and their parents who refused to join in the study were excluded. Consecutive random sampling was used for collecting samples and the sample size was determined by using the sample calculation formula for a cross-sectional study [20]. This study was approved by the Clinical Research Unit at Dr. Soetomo Hospital, Surabaya, Indonesia with the number of 1889/KEPK/III/2020.

### Genetic analysis

**Figure 1.**
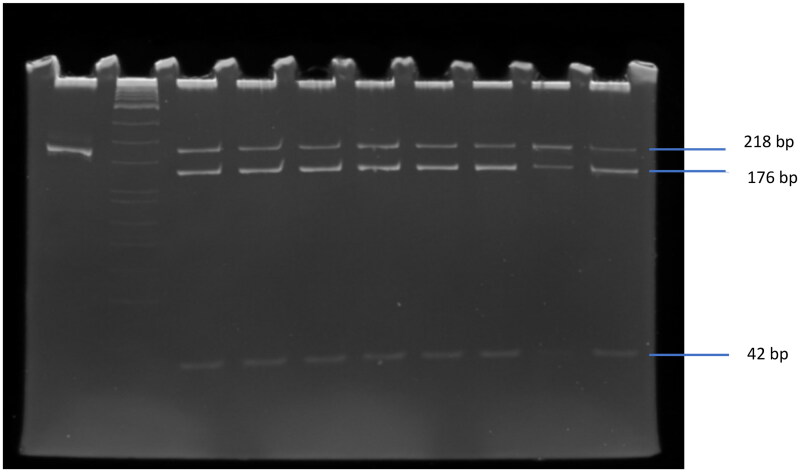
*PTPN22* gene variant C1858T was analysed by PCR-RFLP and visualized by ethidium bromide stained polyacrylamide gel that were line 1 of PCR product without cutting and line 2–9 of samples with cutting by *RsaI* enzyme. M: marker (One STEP Marker 9, Wako, Japan).

The QIAmp DNA Mini Kit was used to extract DNA from peripheral blood mononuclear cells according to standard procedure. The DNA fragment was 218 bp resulted from the amplification of forward primer: 5′-ACTGATAATGTTGCTTCAACGG-3′ and reverse primer: 5′-TCACCAGCTTCCTCAACCAC-3′ by polymerase chain reaction (PCR) (Applied BioSystems, Foster City, CA). The PCR mixture consisted of 10× PCR buffer, 250 ng template DNA, 20 pmol of each primer, 1.5 mM MgCl_2_, 0.2 mM of each of the deoxyribonucleotide triphosphates, and 1 U GoTaq DNA polymerase (Promega, Fitchburg, WI). The amplification consists of denaturation for 2 min at 94 °C followed by 35 cycles at 94 °C for 30 s, annealing 30 s at 60 °C and 30 s at 72 °C, and the final extension for 3 min at 72 °C. The polymorphism of C1858T was identified by restriction fragment length polymorphism (RFLP) as described by Bulut et al. [21]. The PCR product was cut by *RsaI* enzyme (New England Biolabs, Ipswich, MA) at 37 °C for 4 h and resulted in two fragments, 176 bp and 42 bp that indicated the homozygous CC (wild type), whereas the heterozygous CT had three fragments at 218, 176 and 42 bp (see Figure 1). The mutant type (homozygous TT) was at 218 bp that cannot be cut by *RsaI* enzyme. The PCR product, which was not cut by *RsaI* enzyme, was used as the control of 218 bp position.

### Statistical analysis

SPSS version 20.0 (IBM, Armonk, NY) was applied for data analysis. Either Fisher’s exact or Chi-square test was used as requisite for the genotypes polymorphisms and allele distribution comparison between T1DM and control groups. The significant difference or correlation was shown by *p* value (.05).

## Results

Sixty-two children involved in this study were divided as follows: 31 in the T1DM and 31 in the control group. The median age of all subjects was 12.6 (2.7–16.8) years, with 27 males and 35 females, and mostly Javanese tribe (94%, 58/62). The onset of T1DM occurred mostly in the childhood under 12 year-old (90.3%, 28/31) (see Table 1).

**Table 1.** Characteristics of subject.

Characteristics	Case (*n* = 31), *n* (%)	Control (*n* = 31), *n* (%)	Median (min–max)
Age groups			
0–12 years	5 (16.12%)	21 (67.74%)	12.64 (2.74–18.00)
12–18 years	26 (83.87%)	10 (32.25%)	
Sex			
Male	12 (38.70%)	15 (48.38%)	
Female	19 (61.29%)	16 (51.61%)	
Race/tribe			
Javanese	27 (87.09%)	31 (100%)	
Madurese	1 (3.22%)	0 (0%)	
Chinese	1 (3.22%)	0 (0%)	
Malay	2 (6.45%)	0 (0%)	
T1DM onset			9.00 (1.00–16.00)
0–12 years	28 (90.32%)		
12–18 years	3 (9.67%)		

The CC genotype was mostly found in T1DM group but not significantly different in the controls (*p*= .238 95% CI: 0 (0–NA)). The prevalence of CT genotype in T1DM children was 90.3% (28/31), while 100% (31/31) was in the control. The frequency of the C allele and T allele in children with T1DM was 54.8% and 45.2%, respectively (Table 2).

**Table 2.** Polymorphism of *PTPN22* C1858T distribution.

Variables	T1DM (*N* = 31)	Control (*N* = 31)	*p* Value	OR (95% CI)
Genotype				
CC	3 (9.7%)	0 (0%)	.238^a^	0 (0–NA)
CT	28 (90.3%)	31(100%)		
TT	0 (0%)	0 (0%)		
Allele				
C	68 (54.8%)	62 (50%)	.525^b^	0.8235 (0.5–1.3564)
T	56 (45.2%)	62 (50%)		

^a^ Fisher’s exact test.

^b^ Chi-square test.

## Discussion

Genetics and environment contribute to the risk of T1DM. Although the major histocompatibility complex (MHC) is closely connected to the genetic susceptibility to T1DM, the non-HLA gene is also thought to be present to promote the disease. The non-HLA genes such as *PTPN22* may confer the risk of T1DM through T-cell-mediated immune response [22,23].

The *PTPN22* C1858T gene polymorphism in T1DM children was not significantly different in the control group. It seems that *PTPN22* C1858T gene polymorphism is not associated with the occurrence of T1DM. Therefore, interestingly, the variant C allele was present homozygous in 9.7% (3/31) children with T1DM and not in the control group. This might play a role in T1DM susceptibility. Hence, it needs further study with a lot of samples.

Our study was in line with previous studies, which provided no significant association between *PTPN22* C1858T polymorphism and T1DM [22,23]. A recent study from Azerbaijani also reported that polymorphisms of the PTPN22 gene (polymorphisms −1123 C/G and +2740 A/G) did not correlate with T1DM [24]. The same result was shown in a study of Egyptian children with systemic lupus erythematosus [25]. Furthermore, a meta-analysis study that described the *PTPN22* C1858T polymorphism in Europe and the American population may play as a risk factor in T1DM. However, in contrast with this study, *PTPN22* was associated with T1DM in the Colorado, Egyptian children and Asian population [26–28].

This study found genotype-CC and allele-C more frequent in T1DM children. In contrast, another study showed that the TT-1858 genotype was more prevalent in children with T1DM (*p*= .038, OR: 3.16; 95% confidence interval (CI): 1.28–7.09). The study concludes that *PTPN22* C1858T polymorphisms increased the risk factor of T1DM [27]. Other studies also suggest that the prevalence of the *PTPN22* variant (rs2542151), the G allele, may increase the risk of T1DM [28].

The limitation is that this is a single-centre study, further multicentre research is necessary to gather additional information on Indonesian races.

## Conclusions

The homozygous genotype-CC and allele-C are more often found in T1DM. This study shows that *PTPN22* C1858T polymorphism did not significantly had a role in T1DM genetic susceptibility. Further studies, such as multicentre studies and studies in different ethnic groups, are needed for the influence of the *PTPN22* C1858T polymorphism to the T1DM susceptibility.

## Data Availability

The corresponding author can provide you with the information needed to understand the results of this study.
